# A tissue microRNA signature that predicts the prognosis of breast cancer in young women

**DOI:** 10.1371/journal.pone.0187638

**Published:** 2017-11-15

**Authors:** Ai Hironaka-Mitsuhashi, Juntaro Matsuzaki, Ryou-u Takahashi, Masayuki Yoshida, Yutaka Nezu, Yusuke Yamamoto, Sho Shiino, Takayuki Kinoshita, Toshikazu Ushijima, Nobuyoshi Hiraoka, Chikako Shimizu, Kenji Tamura, Takahiro Ochiya

**Affiliations:** 1 Division of Molecular and Cellular Medicine, National Cancer Center Research Institute, Tokyo, Japan; 2 Courses of Advanced Clinical Research of Cancer, Juntendo University Graduate School of Medicine, Tokyo, Japan; 3 Department of Pathology and Clinical Laboratories, National Cancer Center Hospital, Tokyo, Japan; 4 Department of Breast Surgery, National Cancer Center Hospital, Tokyo, Japan; 5 Division of Epigenomics, National Cancer Center Research Institute, Tokyo, Japan; 6 Department of Breast and Medical Oncology, National Cancer Center Hospital, Tokyo, Japan; University of South Alabama Mitchell Cancer Institute, UNITED STATES

## Abstract

Since breast cancers in young women are generally aggressive, young patients tend to be intensively treated with anti-cancer drugs. To optimize the strategy for treatment, particularly in young women, prognostic biomarkers are urgently required. The objective of this study was to identify a tissue microRNA (miRNA) signature that predicts prognosis in young breast cancer patients. Total RNA from 45 breast cancer patients aged <35 years was extracted from formalin-fixed paraffin-embedded (FFPE) tissues and analyzed using miRNA microarrays. Patients were categorized into two groups according to recurrence status within the 5 year period after surgery: recurrence (n = 11) and non-recurrent (n = 34). Histological parameters of hormone receptors and Ki-67 were statistically compared between the two groups. Differentially expressed miRNAs were identified, and their associations with overall survival (OS) were evaluated by log-rank test. The median observation period was 5.8 years for the recurrent group, and 9.1 years for the non-recurrent group. Nine miRNAs were significantly differentially expressed between the recurrent and non-recurrent groups. Receiver Operating Characteristic curve analysis was performed to evaluate the prediction accuracy of the identified miRNAs, and the resultant area under the curve was >0.7. Five of the miRNAs were validated by qRT-PCR, and the expression levels of three of those five (miR-183-5p, miR-194-5p, and miR-1285-5p), both alone and in combination, were associated with OS. In conclusion, we identified three candidate miRNAs that could be used separately or in combination as prognostic biomarkers in young breast cancer patients. This miRNA signature may enable selection of better treatment choices for young women with this disease.

## Introduction

Breast cancer is the most common cancer in women worldwide [1–3]. In the non-Hispanic white population in the West, breast cancer most often occurs in postmenopausal women. However, in parts of the world such as North Africa, the Middle East, and Eastern Asia, a higher proportion of women with breast cancer are diagnosed at ages 50 years or younger.

The onset of breast cancer among young patients is a major clinical issue in cancer etiology. Compared with the elderly, young women (<35 years old) are more often diagnosed with advanced-stage disease [4] and more frequently experience recurrence [5–11]. Several studies reported that breast cancers arising in young women exhibit more aggressive tumor characteristics, including high histological grade (HG), high proliferation rate, high-level lymphovascular invasion, and high rate of estrogen receptor (ER) and/or progesterone receptor (PgR) negativity [4, 5, 7–9, 12]. Consequently, physicians generally offer young breast cancer patients intensive adjuvant and neoadjuvant treatment, often yielding favorable outcomes [4, 13, 14]; however, those treatments are a unique challenge for young patients due to anxiety related to fertility-related issues, and poorer perceived quality of life, resulting in early discontinuation of hormone therapy [15] [16]. In light of these concerns, treatment decision-making in young women requires more extensive consideration [13, 16].

Many patient- and therapy-related factors, including expression of ER and/or PgR and human epidermal growth factor receptor 2 (HER2) amplification status, influence clinical outcome, and are therefore considered as prognostic and predictive biomarkers. Currently, treatment decisions are mainly informed by immunohistochemistry (IHC) assessments of ER, PgR, and HER2 [17, 18]. However, the current diagnosis and treatment settings were developed based on results obtained primarily in postmenopausal women [19, 20], and their ability to predict prognosis in young patients remains unclear. Therefore, novel biomarkers are urgently needed to predict prognosis, as well as optimize treatment strategy, in young women with breast cancer.

Recently, microRNAs (miRNAs), small non-coding RNA molecules that bind to complementary sequences on target messenger RNA transcripts, were proposed as potential prognostic biomarkers and predictors of treatment response in cancer [21, 22]. Deregulation of miRNA expression in breast cancer patients has been detected in both blood samples [23–25] and tumor tissues [25–33]. Formalin-fixed paraffin-embedded (FFPE) tissues are stored for long periods of time, but due to their short length miRNAs are preserved under these conditions without degradation [34], suggesting that a tissue miRNA signature is an empirically feasible source of material for identification of cancer biomarkers.

In this study, we used laser-captured microdissected FFPE samples and performed microarray analysis to obtain tissue miRNA profiles from breast cancers in young women. Microarray data from 45 young breast cancer patients were validated by TaqMan-based quantitative RT-PCR (qRT-PCR). As potential prognostic markers, three miRNAs (miR-183-5p, miR-194-5p, and miR-1285-5p) were identified whose expression levels were significantly altered in breast cancer samples of young women who experienced recurrence in the first 5 years after surgery.

## Materials and methods

### Ethics committee approval

This study was approved by the internal review board of the National Cancer Center, Tokyo, Japan (no. 2014–386).

### Clinical samples

FFPE samples were obtained from breast cancer patients who were admitted or referred to the National Cancer Center Hospital (NCCH) between 2001 and 2010. Signed informed consent to provide tumor tissues for research use was obtained from each patient prior to tissue sample collection. Breast cancer patients (women aged <35 years) with the following characteristics were excluded: (i) previous or current neoadjuvant treatment; (ii) a prior history of cancer; (iii) diagnosis as invasive ductal carcinoma of a special type. Breast tissues were collected according to standard operating procedures at NCCH.

### Histopathological examination and IHC

Paraffin blocks were stored in an environment with controlled temperature and humidity. For IHC, 4 μm sections were cut from each tissue block and stained with hematoxylin and eosin (HE). HE-stained slides were evaluated to select representative tumors and to verify high tumor content. The following antibodies were used for IHC: ER (SP1, rabbit monoclonal primary antibody CONFIRM, Roche Diagnostics, Tokyo, Japan), PgR (1E2, rabbit monoclonal primary antibody CONFIRM, Roche Diagnostics, HER2 (Dako HercepTest, Agilent Technologies, Tokyo, Japan), Ki-67 (MIB-1, Dako monoclonal mouse antibody, Agilent Technologies, Tokyo, Japan). Expression levels of ER and PgR were recorded using the Allred score [35]. Tumors scoring 3 or higher (corresponding to at least 1% positive cells) were considered positive for ER and PgR [17, 36]. HER2 status was considered positive if membranous immunostaining was IHC 3+ or IHC 2+ and HER2 gene amplification was confirmed by dual color in situ hybridization using the Ventana Inform Dual ISH HER2 kit (Roche Diagnostics. Tokyo, Japan) [18]. The Ki-67 labeling index (LI) was calculated by dividing the number of Ki-67–positive tumor cells by the total number of tumor cells. High- and low-LI groups were separated using the mean Ki-67 LI as a cutoff [37].

### Tissue preparation for laser-capture microdissection

Ten consecutive sections (10 μm) of each tumor were prepared on a microtome and mounted onto polyethylene terephthalate membrane slides (Leica Microsystems, Wetzlar, Germany). HE staining was performed before laser-capture microdissection (LCM). Briefly, tissue sections were initially rinsed for 3 minutes with xylene to remove paraffin from tissues, and then washed serially in 100%, 80%, and 70% ethanol for 30 seconds each. The deparaffinized sections were dried overnight, and then either used directly for LCM or stored at 4°C. To isolate tumor compartments, LCM was performed on a Leica LMD6500. The tissue was then collected in 0.5 ml PCR tube caps.

### RNA isolation and quality assessment

Total RNA was obtained from eight 10 μm FFPE sections using the miRNeasy Kit (Qiagen, Crawley, UK). RNA quality was also evaluated with an Agilent 2100 Bioanalyzer (Agilent Technologies, Tokyo, Japan) and a NanoDrop 1000 spectrophotometer (Thermo Fisher Scientific K. K., Kanagawa, Japan).

### Microarray and bioinformatics

Using 250 ng of total RNA for each sample, comprehensive miRNA microarray analysis was performed with the 3D-Gene^®^ miRNA Labeling kit and the 3D-Gene^®^ Human miRNA Oligo Chip ver. 21 (Toray Industries, Inc., Kanagawa, Japan), which was designed to detect 2565 miRNA sequences registered in miRBase release 21 (http://www.mirbase.org/). Individual miRNAs were regarded as present if the corresponding microarray signal was greater than the (mean + 2 × standard deviation) of the negative control signals, of which the top and bottom 5% ranked by signal intensity were removed. To normalize the signals across different microarrays, the global median normalization method was used, and the median of the distribution was set at 25. When the signal level was below the detectable threshold after background subtraction, the level was replaced by the lowest signal intensity of each miRNA on the microarray minus 0.1 on a base-2 logarithmic scale. All microarray data from this study are in concordance with the Minimum Information about a Microarray Experiment (MIAME) guidelines, and are publicly available through the GEO database (GSE 97811).

### qRT-PCR assay for miRNA

Expression levels of nine differentially regulated miRNAs identified in the microarray experiment were tested by TaqMan-based qRT-PCR. The RT-PCR was carried out in 96-well plates using the StepOnePlus^TM^ Real-time PCR system (Applied Biosystems). All reactions were performed in duplicate. All TaqMan MicroRNA assays were purchased from Applied Biosystems. miR-16 was used as an internal control for normalization.

### Statistical analysis

Demographic and histological parameters were compared between the two groups by Pearson’s chi-square test or Student’s *t*-test as appropriate. Covariates considered in Cox proportion regression analyses were as follows: tumor size (binary, tumor size >2 cm or ≤2 cm); nodal status (binary, nodes involved or not); HG (binary, 3 or <3); ER, PgR, and HER2 status (binary, positive or negative); and Ki-67 LI (binary, high or low). Expression levels of miRNAs were compared by *t*-test. For Kaplan–Meier plots, cutoffs between high and low expression levels of prognostic miRNAs were determined from the receiver operating characteristic (ROC) curve. Differences between survival curves were evaluated by log-rank test. A prediction model using multiple miRNAs associated with overall survival (OS) was constructed by logistic regression analysis. The predictive accuracy of this model was also evaluated by ROC curve and Kaplan–Meier plots. Univariable and multivariable Cox proportion regression analyses were performed to identify factors that were independently associated with OS. Statistical analysis were conducted in JMP 10 (SAS Institute Inc. Japan, Tokyo, Japan) and SPSS Statistics Version 21 (IBM SPSS, Armonk, NY, USA) with a two-sided p-value of <0.05 as the threshold for significance.

## Results

### Characteristics of young breast cancer patients

**Fig 1:**
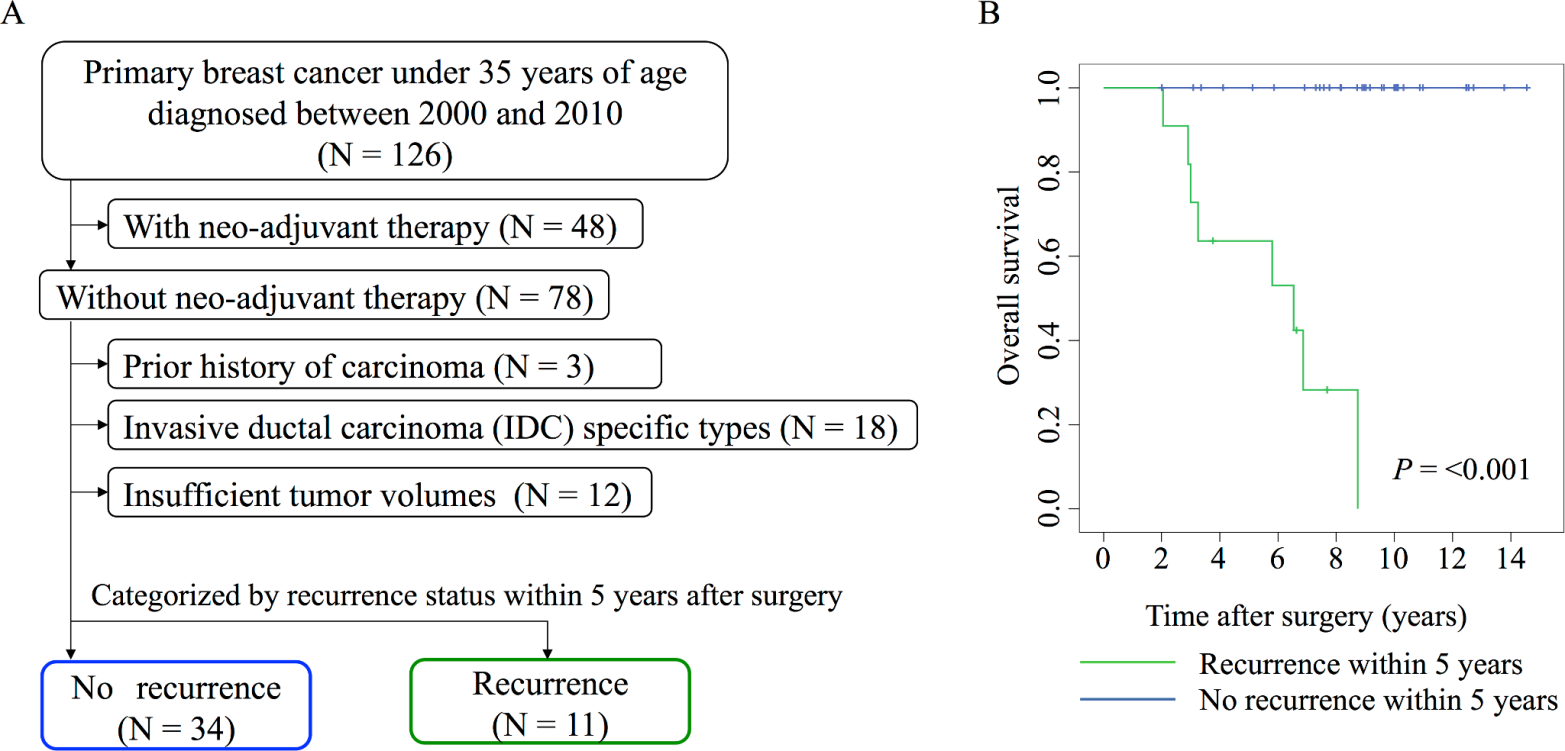
Schematic of selection of candidate miRNA biomarkers of prognosis in young breast cancer patients. (A) Flow diagram showing derivation of the analytic cohort of patients enrolled in this study. (B) Kaplan–Meier plot of recurrent (n = 11) and non-recurrent (n = 34) groups of young breast cancer patients.

Among women aged <35 years who were diagnosed as having invasive breast cancer (n = 126), patients who had undergone neoadjuvant treatment (n = 48), those with a prior history of cancer (n = 3), and those who were diagnosed with invasive breast carcinoma of a special type (n = 18) were excluded. Additionally, patients with insufficient tumor volume, as assessed by routine histological diagnoses (n = 12), were excluded from the experimental cohort (Fig 1A). For the remaining 45 cases, we reviewed the demographic information and clinicopathological characteristics, including invasive tumor size, type, and grade (Tables 1 and 2). To investigate the histological and molecular traits specific to breast cancer with a poor prognosis in young women, we divided the 45 patients into two groups, 11 in the recurrent group and 34 in the non-recurrent group, according to recurrence status within the first 5 years after curative surgery (Fig 1A). The median observation period was 5.8 years (range, 2.1–8.8 years) for the recurrent group and 9.1 years (2.0–14.6 years) for the non-recurrent group. During this period, 13 cases of recurrence were seen: 11 cases of recurrence within 5 years, and two cases of recurrence more than 5 years after surgery. These latter two cases of recurrence were categorized into the non-recurrent group because the recurrence was observed more than 5 years after surgery and the patients did not die. By contrast, the former 11 patients, in whom the recurrences occurred within 5 years, all died during the observation period. This decision was consistent with the overall intent of the classification, which was to stratify the subject population by prognosis (i.e., early recurrent cases were patients with poorer prognoses) (Fig 1B).

**Table 1:** Demographic characteristics of two groups stratified by recurrence status within the 5 year period after surgery.

		Number of patients (%)		
		Recurrence (N = 11)	Non-recurrence (N = 34)	*p* value^†^
Follow-up time (years) [mean (range, median)]		5.2 (2.1–8.8, 5.8)	8.9 (2.0–14.6, 9.1)	<0.001
Age (year) [mean (range)]		31.2 (25–34)	31.5 (22–34)	0.75
BMI (kg/m^2^) [mean ± standard deviation]		20.8 ± 5.5	20.6 ± 2.8	0.93
Menarche (years) [mean (range)]		12.9 (11–15)	12.2 (10–16)	0.11
Smoking status	Current smoker	2 (18)	4 (12)	0.49
	Ex-smoker	3 (27)	5 (15)	
Alcohol consumption	Never	8 (73)	27 (79)	0.64
	the others	3 (27)	7 (21)	
Number of children	0	7 (64)	24 (71)	0.23
	1	2 (18)	5 (15)	
	2	0 (0)	4 (12)	
	3	2 (18)	1 (3)	
Family history of breast cancer		2 (18)	14 (41)	0.17
Family history of the other cancer		7 (64)	22 (65)	0.95
Adjuvant therapy	Not received	1 (9)	2 (6)	0.79
	Hormonal therapy	3 (27)	8 (24)	
	Chemotherpy	4 (36)	9 (26)	
	Hormone + Chemotherapy	3 (27)	15 (44)	
pT factor	pT1	5 (45)	23 (68)	0.16
	pT2	4 (36)	10 (29)	
	pT3	2 (18)	1 (3)	
pN factor	pN0	4 (36)	19 (56)	0.22
	pN1	5 (45)	12 (35)	
	pN2	0 (0)	2 (6)	
	pN3	2 (18)	1 (3)	

†: The P values were calculated by Pearson’s χ^2^ test for categorical variables, and Student’s *t*-test for continuous variables

**Table 2:** Histopathological characteristics of the two groups stratified by recurrence status within the 5 year period after surgery.

			Number of patients (%)		
			Recurrence (N = 11)	Non-recurrence (N = 34)	*p* value^†^
Histological grade of invasive component		1	0 (0)	3 (9)	0.52
		2	3 (27)	11 (32)	
		3	8 (73)	20 (59)	
Tumor infiltrating lymphocytes	Low		4 (36)	11 (32)	0.81
	High		7 (64)	23 (68)	
Lymphovascular invasion	Negative		3 (27)	12 (35)	0.62
	Positive		8 (73)	22 (65)	
Necrosis	Negative		4 (36)	19 (56)	0.26
	Positive		7 (64)	15 (44)	
Comedo necrosis	Negative		5 (45)	16 (47)	0.93
	Positive		6 (55)	18 (53)	
Estrogen receptor	Negative		4 (36)	7 (21)	0.29
	Positive		7 (64)	27 (79)	
Progesterone receptor	Negative		3 (27)	6 (18)	0.49
	Positive		8 (73)	28 (82)	
HER2	Negative		9 (82)	30 (88)	0.59
	Positive		2 (18)	4 (12)	
Ki-67	High		5 (45)	20 (59)	0.44
	Low		6 (55)	14 (41)	
Surrogate subtype	Luminal A-like		3 (27)	17 (50)	0.32
	Luminal B-like (HER2-negative)		4 (36)	8 (24)	
	Luminal B-like (HER2-positive)		1 (9)	4 (12)	
	HER2-enriched		1 (9)	0 (0)	
	Triple-negative		2 (18)	5 (15)	

†: The P values were calculated by Pearson’s χ^2^ test for categorical variables, and Student’s *t*-test for continuous variables

### miRNA microarray analysis of 45 breast cancer samples in young women

**Fig 2:**
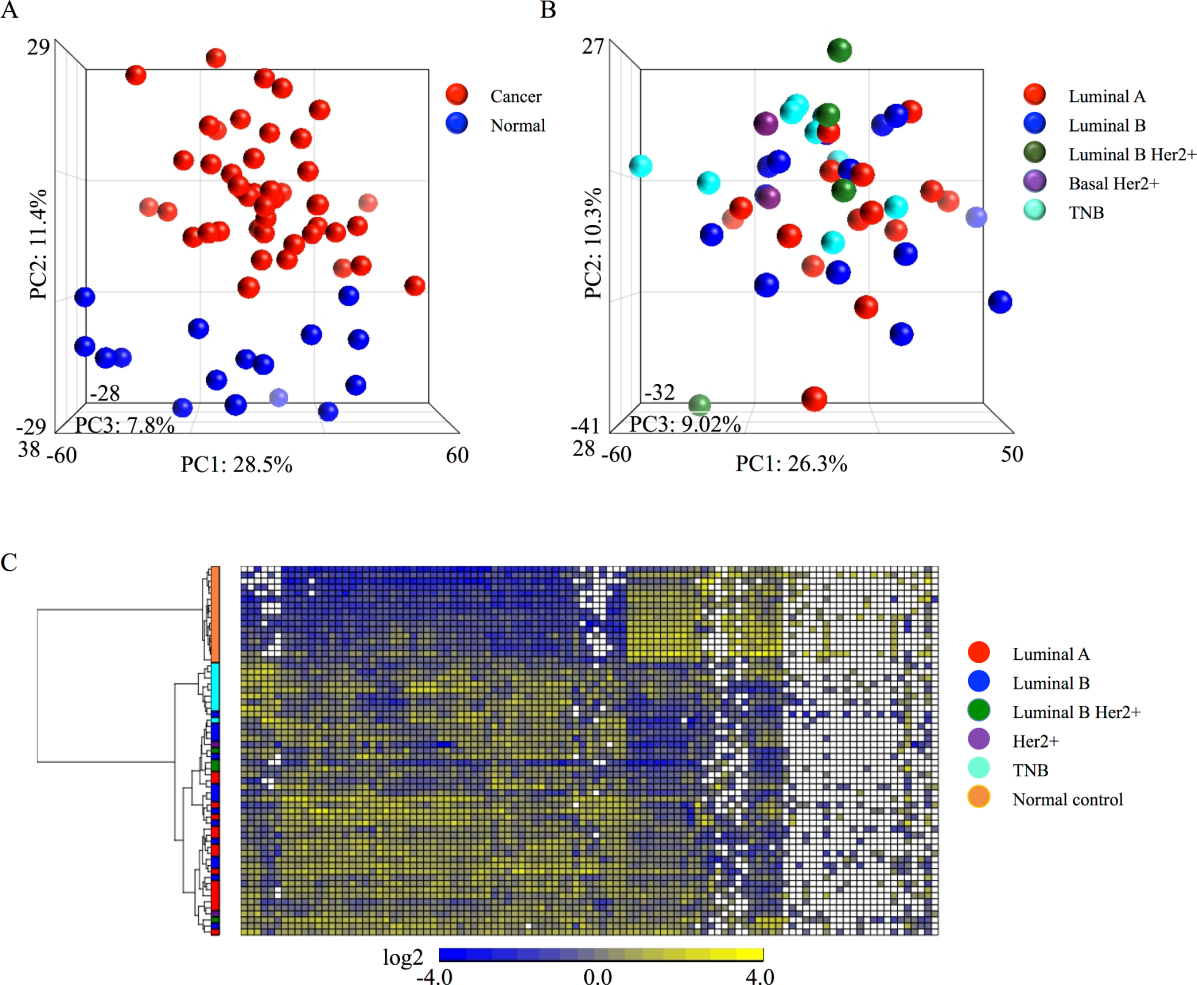
miRNA microarray analysis for 45 breast cancer samples and 16 normal breast epithelial samples. (A) PCA map for 45 breast cancer samples and 16 normal breast epithelial samples. (B) PCA map for 45 breast cancer samples, classified by cancer subtypes. (C) Heatmap showing miRNAs differentially expressed between breast cancer and normal epithelial control samples (fold change ≥2 and p < 0.05; 102 miRNAs).

Using material obtained by LCM from FFPE samples, total RNAs were extracted from 45 breast cancer samples (11 from the recurrence group, 34 from the non-recurrence group) and 16 breast epithelium samples (normal samples) from young women. The samples were subjected to comprehensive miRNA microarray analysis. Based on the principal component analysis (PCA) map, the 45 cancer samples and 16 normal samples were clearly segregated based on the overall miRNA expression profile (Fig 2A). When plotting only cancer samples in the PCA map, the variation in miRNA expression profile among cancer subtypes was not obvious, although there were some weak trends based on differences between the luminal and basal subtypes of breast cancer (Fig 2B). To further investigate the molecular characteristics of the breast cancer samples, we performed hierarchical clustering analysis of the differentially expressed miRNAs (102 miRNAs; fold change ≥2 and p < 0.05 in cancer vs. normal samples). The clustering heatmap revealed more explicit segregation of cancer samples according to their subtypes. In particular, consistent with the miRNA expression profiles in ordinary breast cancer patients, triple-negative breast cancer (TNB) exhibited a distinct miRNA profile from the luminal type (Fig 2C). These data demonstrate that LCM-based miRNA microarray analysis of material FFPE samples can yield accurate expression profiles suitable for examination of tumor properties.

### Searching for prognostic miRNA markers for early recurrence in young women

**Fig 3:**
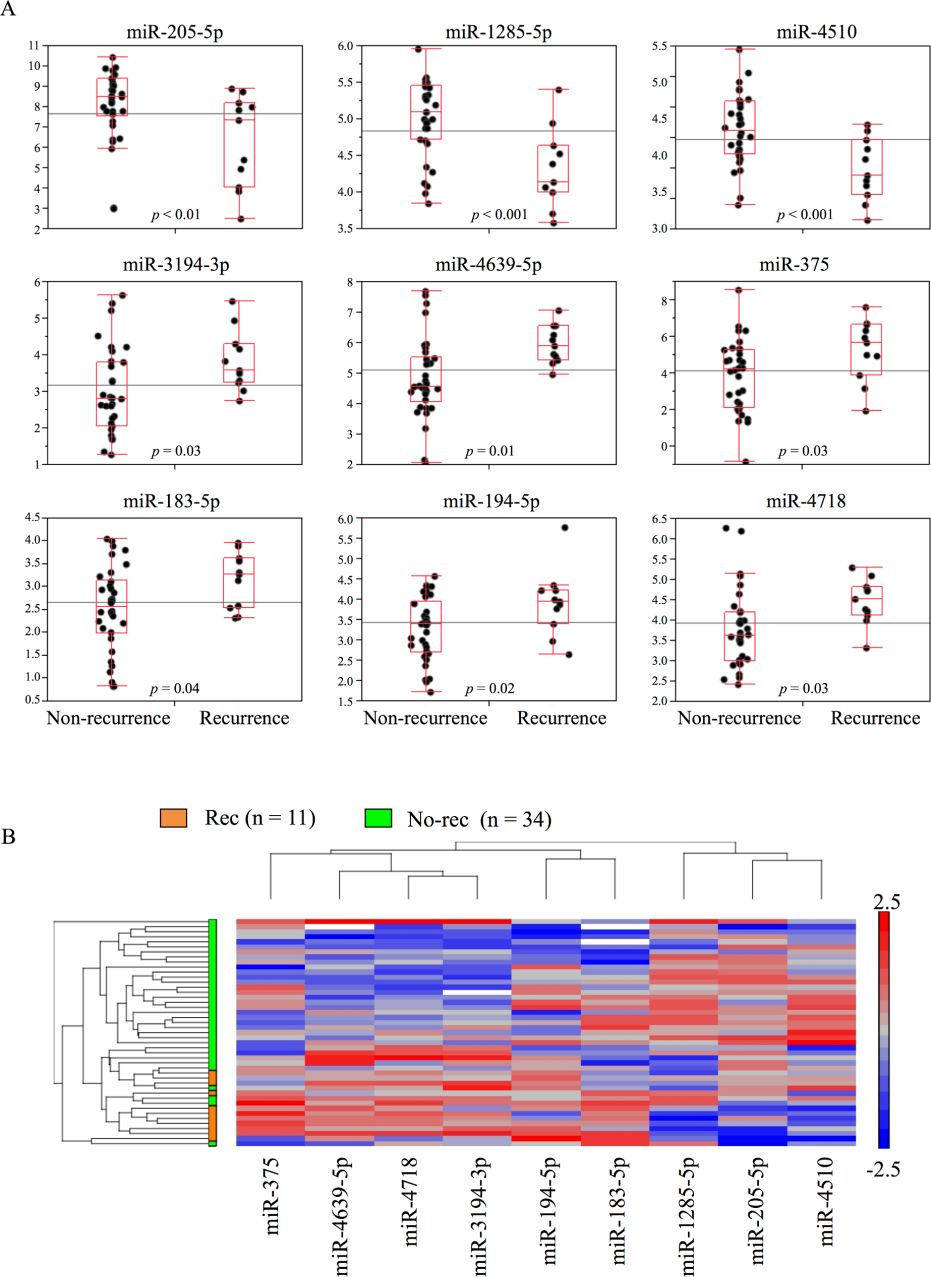
Candidate miRNA selection based on miRNA microarrays. (A) Box plots of nine miRNA candidates with significant differences in expression between recurrence and non-recurrence groups. P-values were calculated using a *t*-test comparing levels between groups, divided according to recurrence status within 5 years after surgery. The middle line indicates the median, the box the interquartile range, and the whiskers the most extreme data point less than 1.5× the interquartile range away from the box. (B) Hierarchical clustering analysis with heatmap for nine candidate miRNAs, considering recurrence status within 5 years after surgery.

To discover prognostic biomarkers of early recurrence in young women, we compared miRNA expression between the recurrent group (n = 11) and non-recurrent group (n = 34). Based on the selection criteria, including fold change >1.5 and average signal level >2^3^, we identified 14 miRNAs (Table 3). Nine of these miRNAs (miR-205-5p, miR-1285-5p, miR-4510, miR-3194-3p, miR-4639-5p, miR-375, miR-183-5p, miR-194-5p, and miR-4718) were expressed at significantly different levels between the recurrent and non-recurrent groups (Student’s *t*-test p < 0.05). Three of the nine (miR-205-5p, miR-1285-5p, and miR-4510) were down-regulated, and the remaining six miRNAs (miR-183-5p, miR-194-5p, miR-4718, miR-3194-3p, miR-4639-5p, and miR-375) were up-regulated, in the recurrent group (Fig 3A). Clustering analysis of the nine miRNAs enabled almost complete segregation of the recurrent and non-recurrent groups (Fig 3B).

**Table 3:** Differentially expressed miRNA between recurrence and non-recurrence groups.

	miR [log_2_]	Non-recurrence (N = 34)	Recurrence (N = 11)	Fold change
*Non-recurrence > Recurrence*	miR-205-5p	8.14	6.35	3.45
	miR-203a-3p	3.9	3.16	1.67
	miR-100-5p	6.61	5.91	1.63
	miR-1285-5p	5.02	4.32	1.63
	miR-4510	4.15	3.48	1.59
	miR-4324	6.02	5.38	1.56
	miR-99a-5p	6.59	5.96	1.55
	miR-487b	5.62	5.03	1.51
*Non-recurrence < Recurrence*	miR-183-5p	2.53	3.15	1.54
	miR-194-5p	3.28	3.93	1.56
	miR-4718	3.77	4.47	1.62
	miR-3194-3p	2.99	3.83	1.79
	miR-4639-5p	4.86	5.94	2.12
	miR-375	3.84	5.27	2.7

### Performance of the prognostic index according to clinical conditions

**Fig 4:**
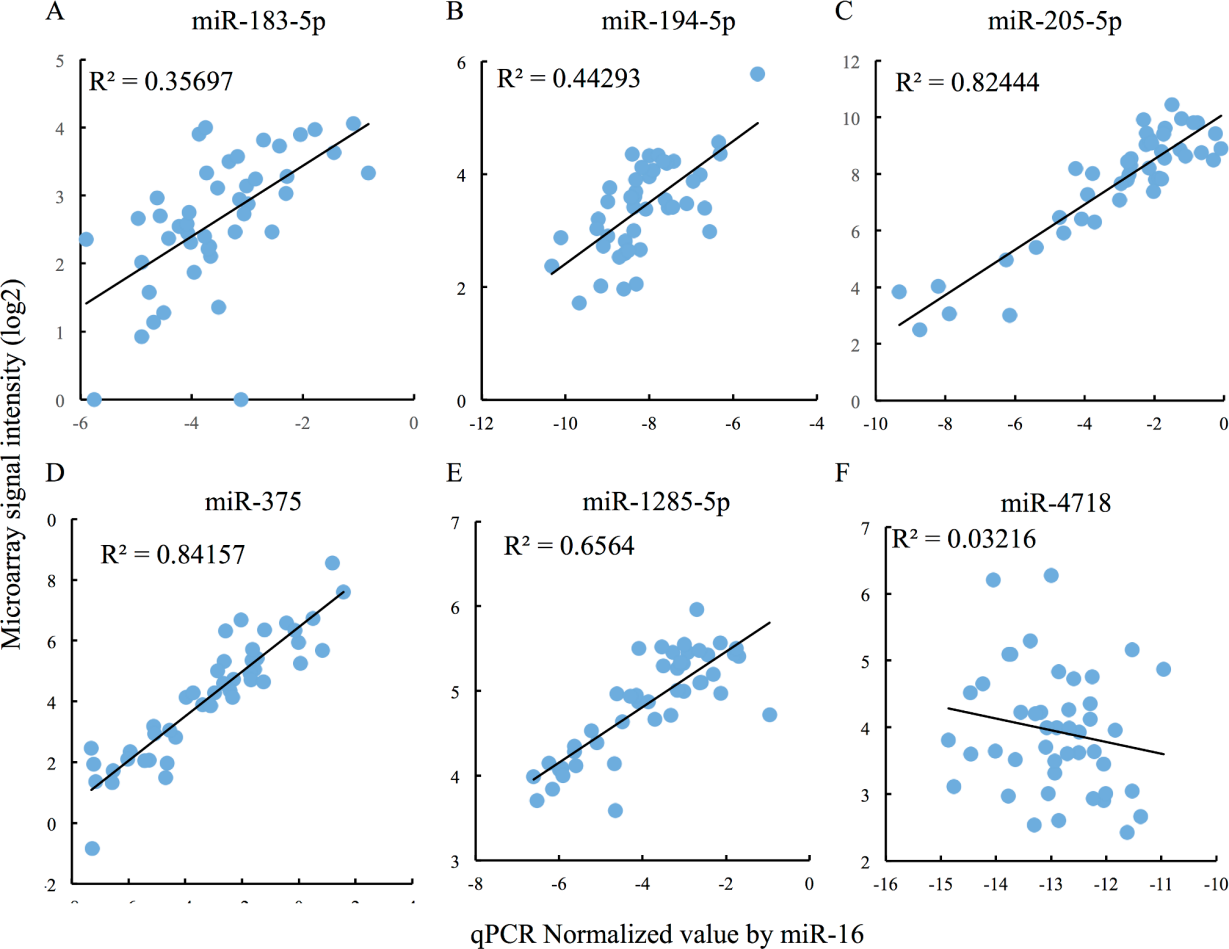
Correlation with quantitative RT-PCR and miRNA microarray data of nine candidate miRNAs. (A–F) Candidate miRNA expression levels were validated by TaqMan-based miRNA qRT-PCR. Average duplicate qPCR data for each miRNA were used as qPCR expression values. Expression of miR-16 was used as an internal control for the normalization. Expression levels measured by qRT-PCR and microarray are shown in scatter plots. R^2^ was calculated to evaluate the correlation.

Before proceeding with further statistical analysis, we performed qRT-PCR to validate the miRNA microarray results. qRT-PCRs were carried out in duplicate, and the mean C_T_ value of each miRNA was normalized against that of miR-16 in the same sample. Five of the nine miRNAs exhibited good correlations between microarray and qRT-PCR results, as demonstrated by scatter plots (Fig 4A–4E). On the other hand, the other four miRNAs, as exemplified by miR-4718 (Fig 4F), exhibited a poor correlation.

**Fig 5:**
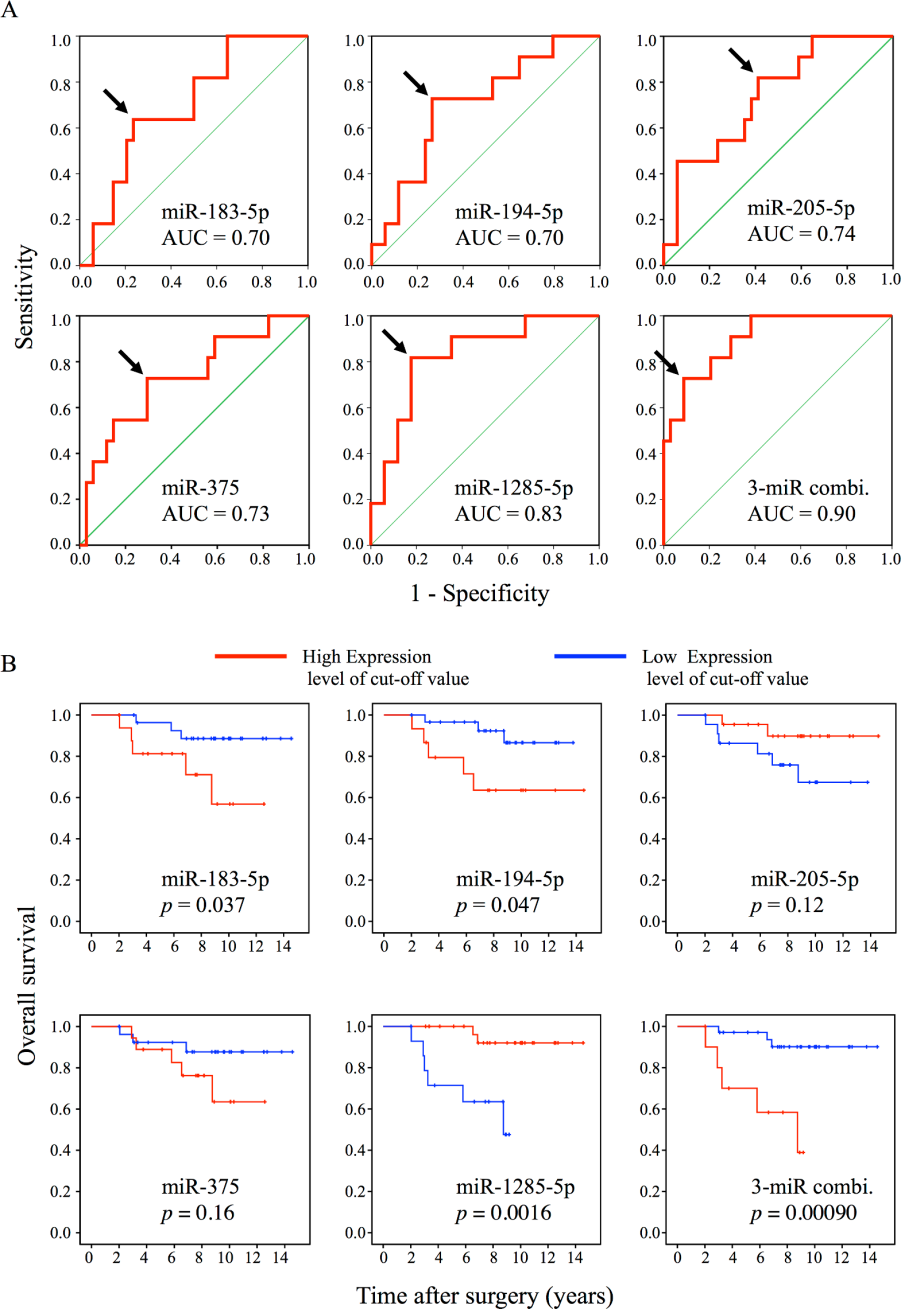
ROC curves and Kaplan–Meier plots for five validated miRNAs (miR-183-5p, miR-194-5p, miR-205-5p, miR-375, and miR-1285-5p). (A) ROC curves of five miRNAs and the three-miRNA combination, with AUC values. Arrows indicate cutoff points. (B) Kaplan–Meier plots of overall survival for five miRNAs and the three-miRNA combination. Based on AUC values and cutoffs, the 45 samples were divided into two groups (high and low expression) for each miRNA, and statistical analysis was performed to calculate p-values using the log-rank test.

Next, we generated ROC curves to evaluate the performance of the five validated miRNAs as biomarkers for the early recurrence of breast cancer in young women. Corresponding AUC and cutoff levels were calculated for the five miRNAs, and all of them had AUC >0.7 (Fig 5A). These cutoff levels were used to perform Kaplan–Meier analysis of OS for each of the five miRNAs, comparing groups of patients with high and low miRNA expression. These analyses confirmed that expression levels of three miRNAs (miR-183-5p, miR-194-5p, and miR-1285-5p) were significantly correlated with patient outcome (Fig 5B). The AUC values for miR-183-5p, miR-194-5p, and miR-1285-5p in discriminating between patients in the recurrent and non-recurrent group were 0.70, 0.70, and 0.83, respectively (Fig 5A). To further emphasize the significance of their prognostic value for early recurrence in young breast cancer patients, we analyzed the three miRNAs in the whole population of breast cancer patients, and found that none of them predicted significant differences in OS (S1 Fig). Thus, these three miRNAs might be selectively effective for predicting prognosis in young patients.

Finally, to maximize prognostic potential, we combined three miRNA levels and generated a ROC curve, resulting in further improvement in AUC (to 0.90) for the prediction of early recurrence (Fig 5A), as well as better OS in the Kaplan–Meier plot (Fig 5B). These results suggest that the prognostic model incorporating the combination of these three miRNAs can predict early recurrence in young breast cancer patients with high accuracy

**Fig 6:**
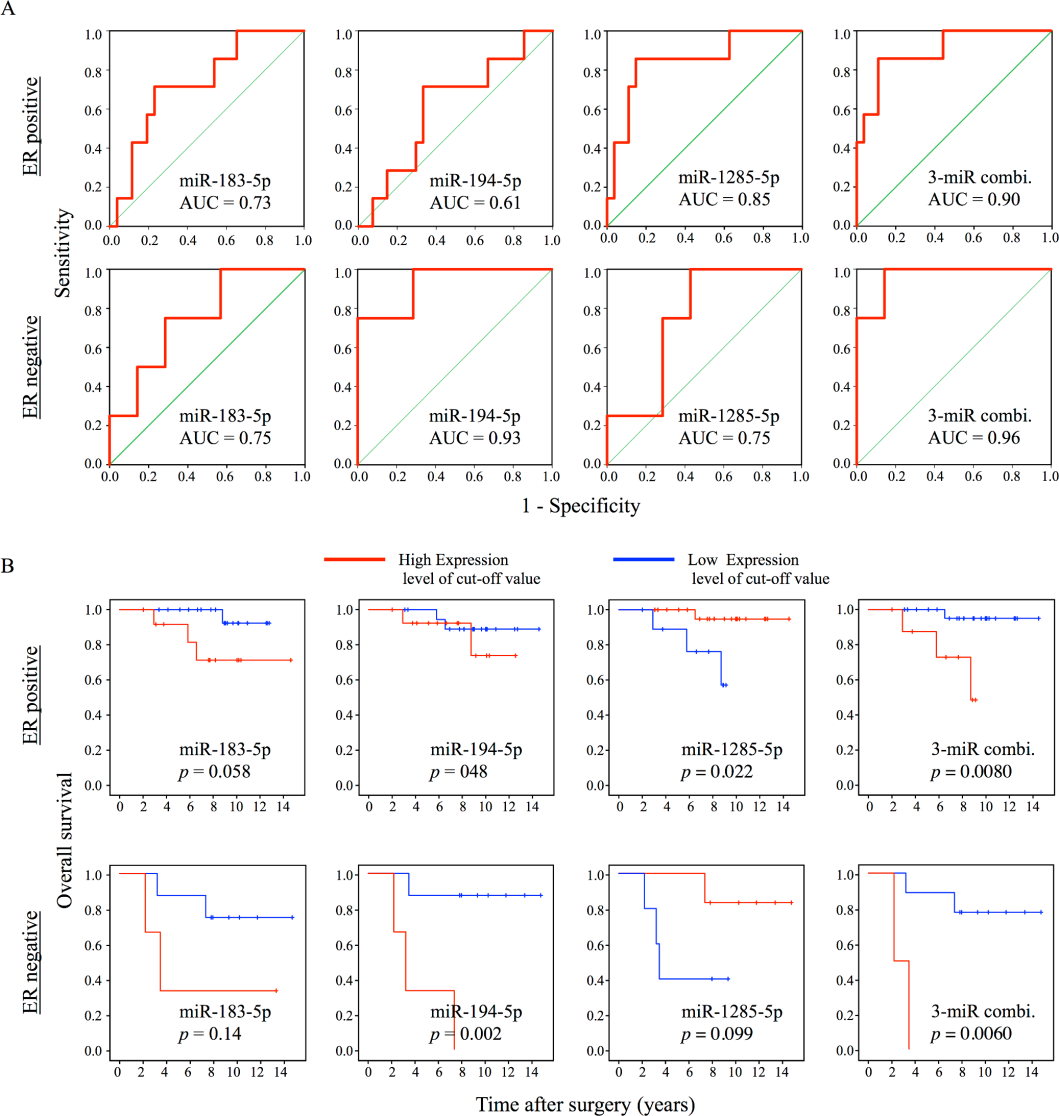
ROC curves and Kaplan–Meier plots for the three validated miRNAs (miR-183-5p, miR-194-5p, and miR-1285-5p) in ER-positive and -negative subgroups. (A) ROC curves of three miRNAs and their combination with AUC values. (B) Kaplan–Meier plots of overall survival for three miRNAs and their combination. Cutoff values for miRNA levels were the same as in Fig 5B. p-values were calculated by the log-rank test.

To further examine the association of OS with miRNA expression levels and histopathological factors, we performed a Cox regression hazard analysis, which revealed that the three miRNAs and ER status were independent predictors (Table 4). The results of subgroup analysis for ER-positive and -negative patients also showed that a combination of three miRNAs (miR-183-5p, miR-194-5p, and miR-1285-5p) accurately predicted early recurrence and were positively associated with overall survival irrespective of ER status (Fig 6).

**Table 4:** Cox regression hazard analysis for overall survival.

		Univariate analysis			Multivariate analysis^†^		
		HR	(95% CI)	*p* value	HR	(95% CI)	*p* value
miR-183-5p	Low	Ref.			Ref.		
	High	4.09	(0.97–17.2)	0.06	7.71	(1.55–38.3)	0.01
miR-194-5p	Low	Ref.			Ref.		
	High	3.88	(0.92–16.4)	0.07	7.07	(1.00–50.1)	0.05
miR-1285-5p	High	Ref.			Ref.		
	Low	8.63	(1.73–43.0)	0.009	8.98	(1.44–56.0)	0.02
T factor	1	Ref.					
	2–3	1.71	(0.43–6.88)	0.45			
N factor	0	Ref.					
	1–3	3.12	(0.63–15.5)	0.17			
Histological Grade	1–2	Ref.					
	3	2.06	(0.42–10.2)	0.38			
ER	Positive	Ref.			Ref.		
	Negative	3.30	(0.82–13.2)	0.09	14.0	(1.78–110.6)	0.01
PgR	Positive	Ref.					
	Negative	2.62	(0.63–11.0)	0.19			
HER2	Negative	Ref.					
	Positive	1.95	(0.39–9.68)	0.41			
Ki-67	Low	Ref.					
	High	1.38	(0.34–5.52)	0.65			

†, factors that were marginally associated with overall survival in univariate analysis (p<0.1) were included in multivariate analysis

ER, estrogen receptor; PgR, progesterone receptor

## Discussion

Unlike previous reports, which assessed prognostic biomarkers for postmenopausal women with breast cancer, this study focused on young patients (aged <35 years). To discover prognostic biomarkers for the early recurrence of breast cancer in young women, we performed miRNA microarray analysis, which identified three miRNAs that were down-regulated (miR-205-5p, miR-1285-5p, and miR-4510) and six that were up-regulated (miR-183-5p, miR-194-5p, miR-4718, miR-3194-3p, miR-4639-5p, and miR-375) in young women with recurrent breast cancer. Five of these miRNAs (miR-183-5p, miR-194-5p, miR-205-5p, miR-375, and miR-1285-5p) were validated by qRT-PCR, revealing that three of them (miR-183-5p, miR-194-5p, and miR-1285-5p) were associated with OS in Kaplan–Meier analysis. Based on our results, we propose that the three-miRNA combination could serve as an accurate prognostic biomarker for prognosis in young breast cancer patients.

Generally, miRNAs play oncogenic or tumor-suppressive roles in a context-dependent manner. miR-183-5p is a member of the miR-183 family, which also includes miR-183, miR-96, and miR-182; these three miRNAs have homologous sequences and are polycistronically expressed. Aberrant expression of the miR-183/-96/-182 cluster is associated with aggressiveness in multiple cancers, including breast cancer [32, 38, 39]. Elevated expression of miR-183-5p suppresses cell migration in the T47D cell line, suggesting that miR-183-5p inhibits metastatic potential [40]. By contrast, several lines of evidence indicate that the miR-183/-96/-182 cluster plays oncogenic roles in breast cancer [32, 33, 38, 39]. Transcription of the cluster is regulated by *ZEB1* and *HSF2* in breast cancer cell lines [39]. Inhibition of miR-183-5p and miR-182-5p *in vitro* causes up-regulation of the tumor-suppressive genes *CBX7* and *EGR1* [33]. miR-183-5p targets *PDCD4*, resulting in cell proliferation and inhibition of apoptosis [38], which is in support of our results.

In contrast to miR-183-5p, the biological function and potential molecular mechanisms of miR-194-5p remain unknown. In breast cancer, miR-194-5p is specially induced by trastuzumab, a humanized murine anti-HER2 monoclonal antibody, and inhibits invasion and migration of HER2-overexpressing breast cancer cells by targeting Talin 2 [41]. In prostate cancer, high expression of miR-194-5p in tumor tissues is associated with poor prognosis [42], whereas the reverse relationship is seen in hepatocellular [43] and renal cell carcinoma [44]. Our results provide the first evidence of a correlation between the expression level of miR-194-5p and clinical outcomes in breast cancer. Previously, however, Huo et al. reported high expression of miR-194-5p as a circulating miRNA signature in blood associated with recurrence [23].

To our knowledge, the role of miR-1285-5p in breast cancer has not been previously reported, although a few studies have mentioned miR-1285-3p in the context of renal cell and hepatocellular carcinoma [44]. Here, we observed aberrant expression of miR-1285-5p in breast cancer with recurrence, particularly in young women. The biological function and potential molecular mechanisms of these miRNAs need to be elucidated. Also, future studies should validate the prognostic values of the expression levels of these miRNAs.

Although we did not detect an association of miR-205-5p or miR-375 with clinical outcome, these miRNAs have been implicated in breast cancer. Expression of miR-205-5p is significantly reduced in tumor tissues in comparison with neighboring normal tissues, as documented by other groups [25–27, 45]. In breast tissues, miR-205-5p is expressed at high levels in myoepithelial and basal epithelial cells in normal breast [45], and retention of miR-205-5p expression in cancer cells is associated with better clinical outcomes [27, 28]. On the other hand, miR-375 is up-regulated in ER-positive cell lines and plays a role in regulating ER [46]. Low expression of miR-375 was proposed as a predictor of relapse by Hoppe et al., who examined the associations between differentially expressed miRNAs, ER status, and tamoxifen resistance in postmenopausal women [30]. By contrast, Zehentmary reported high expression of miR-375 as a predictor of local recurrence of early breast cancer [29]. Additionally, miR-375 modulates sensitivity to tamoxifen [47] and trastuzumab [48] in breast cancer cells. Taking into consideration the influence of miR-375 on treatment resistance, it remains unclear whether this miRNA would have prognostic or predictive values in breast cancer in young women.

This study had several limitations. 1) Only a retrospective analysis was conducted in a single institute. 2) Our research cohort was not large because young women aged <35 years with breast cancer are rare, accounting for only 2.7% of all breast cancers in Japan, and most of these patients receive neoadjuvant therapies [4]. Especially, the sample size of the patients with a poor prognosis, such as those that were HER2-enriched and triple negative, was small. Therefore, the statistical significance for the association between the identified miRNAs and poor prognostic subtypes was insufficient. 3) We could not compare expression levels between paired cancer and normal tissues. Consideration of these points is essential for identifying the ideal biomarkers for diagnosis of breast cancer for use in early detection. However, the main goal of this study was identification of prognostic biomarkers, as well as biomarkers for the optimal treatment choices, in young women who were already diagnosed with breast cancer. In other words, although some concerns exist regarding sample collection, by choosing a research cohort with the same treatment background and simply comparing recurrence status, we identified candidate prognostic biomarkers for young breast cancer patients.

The distinct expression profiles of miRNAs in cancer, especially those that differ according to tumor status, have received a great deal of attention as both prognostic and diagnostic biomarkers. To discover prognostic markers for early recurrence in young women, we performed comprehensive miRNA microarray analysis of FFPE samples from young breast cancer patients by comparing recurrence and non-recurrence status. These results demonstrated that three miRNAs (miR-183-5p, miR-194-5p, and miR-1285-5p), used in combination, could be used to accurately predict early recurrence. Because anti-cancer drugs for young women must be selected carefully, judgments based on the miRNA signature may provide better treatment choices for these patients. In summary, our data provide insight into the potential use of miRNA expression profiles for predicting prognosis in young breast cancer patients.

## Supporting information

S1 Fig Kaplan–Meier plots of three miRNAs in the overall population of breast cancer patients using public databases. Kaplan–Meier plots were generated using the website http://kmplot.com/analysis/index.php?p=service&cancer=breast_mirna. High- and low-expression groups were divided based on the median value of the expression levels: miR-183-5p (high: n = 631 and low: n = 631), miR-194-5p (high: n = 632 and low: n = 630), miR-1285-5p (high: n = 631 and low: n = 631). Y axis indicates OS.
(TIFF)
